# Early and late-onset colorectal cancers: a case-case comparison of risk factors

**DOI:** 10.1007/s10552-026-02208-2

**Published:** 2026-07-11

**Authors:** Samantha Rees, Rachel Pearlman, Electra D. Paskett, Peter G. Shields, Heather Hampel, Jo L. Freudenheim

**Affiliations:** 1 Department of Epidemiology and Environmental Health, School of Public Health and Health Professions, University at Buffalo, Buffalo, NY USA; 2 Department of Internal Medicine, The Ohio State University Wexner Medical Center, Columbus, OH USA; 3 Division of Cancer Prevention and Control, College of Medicine, The Ohio State University, Columbus, OH USA; 4 The Ohio State University Comprehensive Cancer Center and James Cancer Hospital and Solove Research Institute, Columbus, OH USA; 5 Department of Medical Oncology and Therapeutics Research, City of Hope, Duarte, CA USA

**Keywords:** Early-onset colorectal cancer, epidemiology, case-case comparison study, risk factors

## Abstract

**Purpose:**

Examine which demographic, lifestyle, and clinical risk factors differ between patients with early-onset colorectal cancers (EOCRC) compared to late-onset colorectal cancers (LOCRC).

**Methods:**

We conducted a case-case comparison of risk factors and symptoms for EOCRC and LOCRC, utilizing the Ohio Colorectal Cancer Prevention Initiative (OCCPI) data, a statewide study of newly diagnosed CRC among Ohio residents, aged 20–92 years. Unconditional logistic regression (odds ratios (OR) and 95% confidence intervals (CI)) was used to compare risk factors by age-at-diagnoses; those diagnosed < 50 years (EOCRC, *N* = 288) compared to those diagnosed ≥ 50 years (LOCRC, *N* = 1,018), adjusting for sex, race/ethnicity, education, smoking status, and family history of CRC.

**Results:**

Compared to LOCRC, EOCRC cases had higher odds of ever consuming alcohol (OR = 2.47, CI: 1.55–3.91), consuming more alcohol in their teens/twenties than in other decades (OR = 1.85, CI: 1.36–2.51), and binge drinking (OR = 3.15, CI: 2.31–4.30). EOCRC cases were more likely to have Lynch syndrome (OR = 4.61, CI: 2.72–7.84), and report experiencing pre-diagnostic CRC symptoms (OR = 6.08, CI: 3.77–9.82), including blood in stool (52.3 vs. 30.7%), change in bowel habits (37.2 vs. 19.8%), and bowel obstruction (13.9 vs. 7.7%). Birth weight, inflammatory bowel disease, irritable bowel syndrome, and sex did not differ by age-at-diagnosis. However, when birthweight was compared between the youngest EOCRC to the oldest LOCRC cases (< 40 vs. ≥ 65 years), odds of birthweight of < 6lbs was higher in the youngest cases (OR: 2.15, CI: 0.95–4.87).

**Conclusion:**

Alcohol consumption, consuming the most alcohol in one’s teens/twenties, binge drinking, having Lynch syndrome, and pre-diagnostic CRC symptoms were more associated with EOCRC than LOCRC.

**Supplementary Information:**

The online version contains supplementary material available at 10.1007/s10552-026-02208-2.

## Introduction

In contrast to a decline in colorectal cancer (CRC) incidence in older adults since the mid-1990s by 1–3% annually, there has been roughly a 2% yearly increase in early-onset colorectal cancers (EOCRC, defined as age < 50) in the US during the same period [1–3]. Currently, it is estimated that 13% of all CRC diagnoses are EOCRC [4]. Additionally, the CRC mortality rate in younger individuals has increased by roughly 1% annually from 2008 to 2017 [1]. Reasons for the increase in EOCRC are not clear, limiting detection of high-risk younger individuals who would benefit from targeted interventions. There has been speculation that the increase in EOCRC incidence resulted from changes in screening practices and/or disease reclassification (i.e., tumors of the appendix being reclassified as a CRC), but these theories are debated, and do not likely explain the total increase [5–8]. Incidence rates also increased in Canada, Australia, and the United Kingdom in this younger age group where guidelines differ, making it less likely that the increase in EOCRC is solely the result of changes in screening practices [9–11].

While genetic factors likely play a larger role in EOCRC than in late-onset colorectal cancer (LOCRC), the majority of cases in both groups are among those without either a known genetic etiology or a family history of CRC [12–14]. Researchers have suggested several modifiable and non-modifiable risk factors, including obesity, diabetes, alcohol, diet, and microbiome changes, that may be associated with EOCRC[9, 15–18], but evidence is insufficient to support substantive changes in prevention. To our knowledge, there have not been detailed comparisons of early-life exposures between EOCRC and LOCRC cases [2, 9, 11, 18]. Early-life exposures may be more associated with EOCRC development than LOCRC given the closer temporal proximity to disease onset [19].

With the increasing rates of EOCRC and consequences of later stage diagnoses, there is a critical need to understand the etiology and risk factors associated with EOCRC to inform the identification of high-risk younger individuals [2, 11, 17, 20]. We conducted a case-case comparison of EOCRC and LOCRC cases in the Ohio Colorectal Cancer Prevention Initiative (OCCPI), comparing both early-life and adulthood exposures. Our study offers hypothesis-generating insights, laying the groundwork for future prospective or mechanistic studies.

## Methods

### Study design and population

The OCCPI focused on the reduction of CRC morbidity and mortality across urban and rural communities of Ohio [21]. Approximately 3,471 patients were recruited between 2013 and 2016. Individuals were eligible to participate in the OCCPI if they were: newly diagnosed with colorectal adenocarcinoma with sufficient tumor available to perform mismatch repair (MMR) testing. Individuals were excluded who were ≤ 18 years old, whose tumor was not a colorectal adenocarcinoma after pathological review, whose tumor was insufficient for testing, who were missing a blood sample, or whose diagnosis was made outside of Ohio [21]. Our study included participants who consented to future research and completed the baseline epidemiologic questionnaire (*N* = 1,579). We further excluded 24 participants whose age at CRC diagnosis was not consistently recorded and 273 participants with missing covariate data (including sex, race/ethnicity, education, health insurance status, smoking status, body mass index (BMI), alcohol consumption, and prior CRC screening); the final analytic sample was 1,306 participants (288 EOCRC and 1,018 LOCRC cases).

### Risk factor measures

The OCCPI questionnaire included questions regarding the participants’ CRC diagnosis, demographics, personal and family medical histories, lifestyle and environmental factors. Participants completed an online self-administered questionnaire. Sociodemographic, clinical, and lifestyle factors were self-reported. Our focus included risk factors and symptoms previously found to be associated with LOCRC and all early-life exposures queried. Detail regarding how these risk factors were assessed is included in the *Supplemental Materials, Table* 1.

**Table 1:** Baseline participant characteristics: early-onset and late-onset CRC cases in the Ohio Colorectal Cancer Prevention Initiative (*N* = 1,579)

Risk factor	Early-onset CRC *N* = 323	Late-onset CRC *N* = 1,256	*p*-value^a^
	No. (%)		
Age at diagnosis, mean ± SD	42.9 ± 5.8	64.9 ± 9.4	< .0001
Sex			0.30
Female	170 (52.6)	620 (49.4)	
Male	153 (47.4)	635 (50.6)	
*Missing*	0	1 (0.1)	
Educational attainment			< .0001
≤ High school graduate	90 (27.9)	499 (39.7)	
Some college/technical school/associate degree	112 (34.7)	410 (32.6)	
≥ College degree	118 (36.5)	340 (27.1)	
*Missing*	3 (0.9)	7 (0.6)	
Health insurance^b^			0.37
Insured	314 (97.2)	1,227 (97.7)	
Uninsured	5 (1.55)	12 (1.0)	
*Missing*	4 (1.2)	17 (1.4)	
Race			0.27
White	293 (91.7)	1,167 (92.9)	
Non-white or multi-race	28 (8.7)	87 (6.9)	
*Missing*	2 (0.6)	2 (0.2)	
History of CRC screening			< .0001
No	236 (73.1)	595 (47.4)	
Yes	70 (21.7)	599 (47.7)	
*Missing*	17 (5.3)	62 (4.9)	
BMI (kg/m^2^), mean ± SD	29.4 ± 6.6	28.8 ± 6.1	0.72
Pre-diagnostic symptoms			< .0001
Yes	284 (87.9)	845 (67.3)	
Polyp history			< .0001
No	272 (84.2)	817 (65.1)	
Yes	33 (10.2)	356 (28.3)	
*Missing*	18 (5.6)	83 (6.6)	
Family history of CRC			0.004
Yes	38 (11.8)	232 (18.5)	
Lynch syndrome			< .0001
Yes	35 (10.8)	46 (3.7)	
Diabetes^c^			< .0001
Yes	23 (7.1)	251 (20.0)	
High cholesterol^c^			< .0001
Yes	49 (15.2)	536 (42.7)	
Irritable bowel syndrome^c^			0.40
Yes	13 (4.0)	65 (5.2)	
Inflammatory bowel disease^c^			0.94
Yes	9 (2.8)	34 (2.7)	
Birth Weight			0.73
< 6 lbs	27 (8.4)	104 (8.3)	
6–8 lbs	186 (57.6)	656 (52.2)	
8–10 lbs	63 (19.5)	239 (19.0)	
> 10 lbs	6 (1.9)	34 (2.7)	
*Missing*	41 (12.7)	223 (17.8)	
Smoking status			< .0001
Current	31 (9.6)	112 (8.9)	
Former	88 (27.2)	555 (44.2)	
Never	202 (62.5)	586 (46.7)	
*Missing*	2 (0.6)	3 (0.2)	
Alcohol intake			0.01
Ever consumed	294 (91.0)	1,073 (85.4)	
Never consumed	29 (9.0)	179 (14.3)	
*Missing*	0	4 (0.3)	
Decade with most alcohol Consumption^d^			< .0001
Teens/twenties	206 (70.1)	546 (50.9)	
Other decades	83 (28.2)	424 (39.6)	
*Missing*	5 (1.7)	102 (9.5)	
Binge drinking, during decade with most consumption^e^			< .0001
Ever engaged	192 (59.4)	493 (39.3)	
Never engaged	121 (37.5)	716 (57.0)	
*Missing*	10 (3.1)	47 (3.7)	
Binge drinking, during year Before CRC diagnosis^e^			< .0001
Ever engaged	117 (36.2)	210 (16.7)	
Never engaged	189 (58.5)	983 (78.3)	
*Missing*	17 (5.3)	63 (5.0)	

CRC = colorectal cancer; SD = standard deviation

^a^*p*-values: chi-square tests (categorical) or *t*-tests (continuous); calculations do not include missing values

^b^Insured includes those with private insurance, Medicare, Medicaid, and other

^c^Self-reported disease status, “Has a provider ever told you that you had the following conditions?”

^d^Lifetime alcohol abstainers were not included in this analysis (*N* = 192)

^e^Binge drinking defined as reported consumption of ≥ 4 alcoholic drinks in a 2-hour period for both men and women

### Outcome measure

Age at diagnosis (years), determined from pathology reports, was the primary study outcome. We categorized age at diagnosis as < 50 years old (EOCRC) or ≥ 50 years old (LOCRC) based on the CRC screening guidelines during the study period [22].

### Statistical analysis

We compared participant characteristics of EOCRC cases with LOCRC cases using frequencies and percentages for categorical variables and means/medians and standard deviations (SD) for continuous variables [20, 23–25]. Two variables were not included in further analyses: insurance status because of insufficient variation and household income because of large amounts of missing data for this variable. All other risk factors were moved forward to the next phase of analysis to avoid conditioning on univariable *p*-values.

Unconditional logistic regression was utilized to estimate odds ratios (OR) and 95% confidence intervals (95%CI) for case-case comparisons of EOCRC and LOCRC. All models were adjusted for: sex (female, male), race/ethnicity (non-white/multi-race/ethnicity, white), educational attainment (≤ high school, some college/ technical school/associate degree, ≥ college degree), smoking status (current, former, never), and family history of CRC (yes, no). As a sensitivity analysis, we conducted model-specific covariate adjustments based on individual directed acyclic graphs per risk factor (*Supplemental Materials, Table 2*). We also restricted the analytic sample to those without history of prior CRC screening and to those without a family history of CRC or Lynch syndrome, to examine likely sporadic cases. In addition, we examined associations across four age categories: ≤ 40, 40–50, 50–65, and ≥ 65 years old. We conducted bivariate descriptions of the four CRC age groups and crude multinomial logistic regression analyses to compare the youngest EOCRC to the oldest LOCRC cases; sample size did not allow for covariate adjustment. Lastly, as an exploratory analysis, tumor characteristics were compared between EOCRC and LOCRC cases.

## Results

Participant characteristics by EOCRC and LOCRC status are shown in Table 1. Overall, the sample was predominately white and insured. Clinical factors including CRC symptoms before diagnosis (87.9 vs. 67.3%) and Lynch syndrome (10.8 vs. 3.7%) were reported more frequently by EOCRC cases. Family history of CRC (11.8 vs. 18.5%) and diabetes (7.1 vs. 20.0%) were less common among EOCRC compared to LOCRC. Ever consuming alcohol (91.0 vs. 85.4%), consuming more alcohol in their teens/twenties compared to other decades (70.1 vs. 50.9%), engaging in binge drinking in the year before CRC diagnosis (36.2 vs. 16.7%) and never smoking (62.5 vs. 46.7%) were reported more by those with EOCRC than LOCRC. The two groups did not differ by sex, race/ethnicity, BMI, irritable bowel syndrome (IBS), inflammatory bowel disease (IBD), or birth weight. EOCRC cases were more likely to present with cancer in the left colon (40.0 vs. 22.5%) or rectum/rectosigmoid (38.4 vs. 28.4%) than LOCRC cases, who were more likely to experience CRC in the right (21.1 vs. 35.5%) or transverse colon (5.0 vs. 7.5%) (Supplemental Materials, Table 3).

Specific CRC symptoms were compared in Table 2. Blood in stool (52.3 vs. 30.7%), change in bowel habits (37.2 vs. 19.8%), and bowel obstruction (13.9 vs. 7.7%) were more likely to be reported by EOCRC than LOCRC. We saw no difference in reports of anemia and other symptoms between EOCRC and LOCRC. Though not statistically significant, pre-diagnostic weight loss was reported somewhat more often by those with EOCRC (9.6 vs. 6.7%).

**Table 2:** Comparison of self-reported pre-diagnostic CRC symptoms between early-onset CRC and late-onset CRC cases in the OCCPI (*N* = 1,579)

Pre-diagnostic symptoms	Early-onset CRC	Late-onset CRC	*p*-value^a^
	*N* = 323	*N* = 1,256	
	No. (%)		
Any symptoms			< .0001
No	39 (12.1)	411 (32.7)	
Yes	284 (87.9)	845 (67.3)	
Blood in stool			< .0001
No	154 (47.7)	871 (69.4)	
Yes	169 (52.3)	385 (30.7)	
Anemia			0.82
No	283 (87.6)	1,106 (88.1)	
Yes	40 (12.4)	150 (11.9)	
Weight loss			0.07
No	292 (90.4)	1,172 (93.3)	
Yes	31 (9.6)	84 (6.7)	
Change in bowel habits			< .0001
No	203 (62.9)	1,008 (80.3)	
Yes	120 (37.2)	248 (19.8)	
Bowel obstruction			0.001
No	278 (86.1)	1,159 (92.3)	
Yes	45 (13.9)	97 (7.7)	
Other symptoms			0.97
No	278 (86.1)	1,082 (86.2)	
Yes	45 (13.9)	174 (13.9)	

OCCPI = Ohio colorectal cancer initiative; CRC = colorectal cancer

^a^*p*-values: chi-square tests (categorical) or t-tests (continuous); calculations do not include missing values

**Fig. 1:**
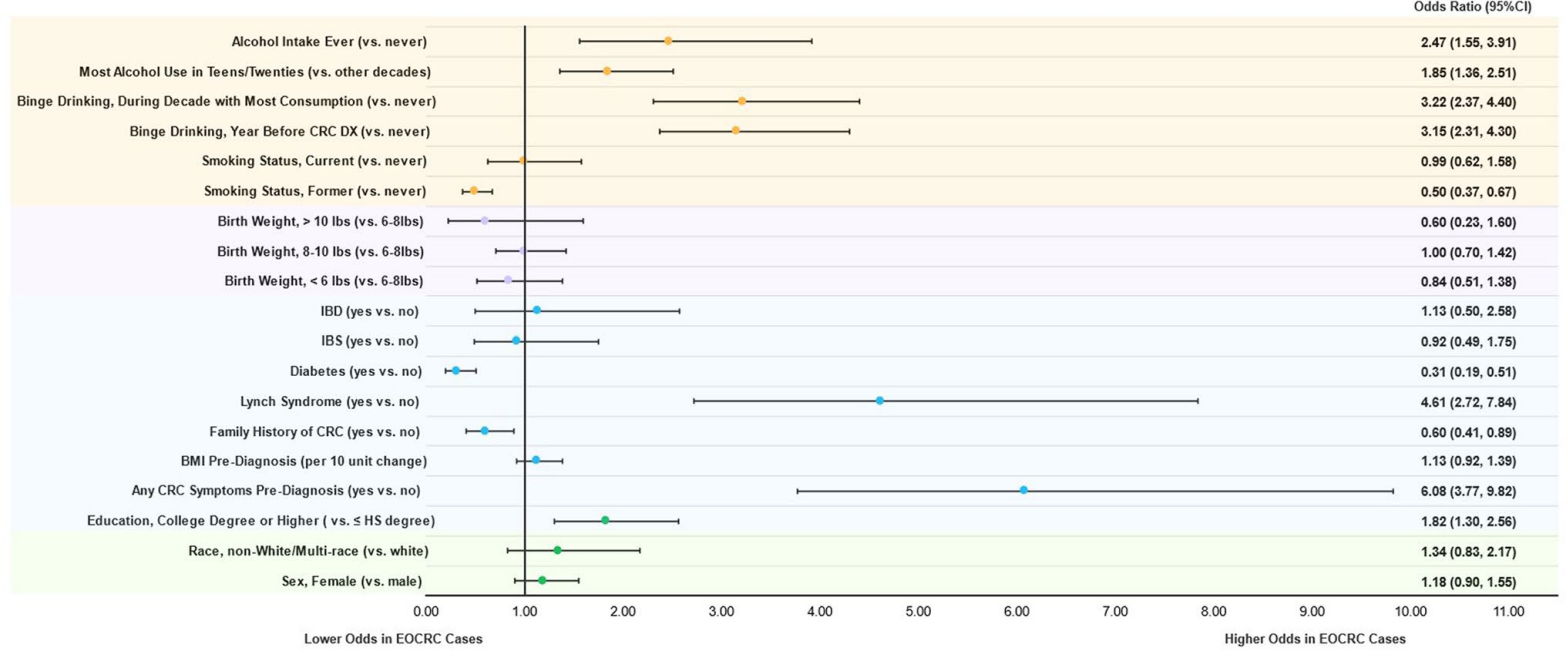
Comparisons of risk factors and symptoms associated with EOCRC cases to LOCRC cases (*N* = 1,306 total; 288 EOCRC and 1,018 LOCRC cases). Forest plot of adjusted logistic regression models of potential risk factors and symptoms comparing EOCRC to LOCRC (referent group: LOCRC). Sociodemographic factors are shown in green, clinical factors in blue, birth weight in purple, and lifestyle factors in orange. All models adjusted for sex, race, education, smoking status, and family history of CRC. OR = odds ratios, 95%CI = 95% confidence intervals

In Fig. 1, multivariable results are presented comparing the odds of a characteristic for EOCRC to LOCRC cases, adjusted for sex, race/ethnicity, educational attainment, smoking status, and family history of CRC. Those with EOCRC were more likely than those with LOCRC to report ever consuming alcohol (OR = 2.47; 95%CI = 1.55–3.91), to have consumed the most alcohol in their teens/twenties compared to other decades of life (OR = 1.85; 95%CI = 1.36–2.51), and to have engaged in binge drinking the year before diagnosis and/or when they were consuming the most alcohol (OR = 3.15; 95%CI = 2.31–4.30 and OR = 3.22; 95%CI = 2.37–4.40, respectively). EOCRC patients were more likely to report any CRC symptoms before diagnosis (OR = 6.08; 95%CI = 3.77–9.82), Lynch syndrome (OR = 4.61; 95%CI = 2.72–7.84), and educational attainment of a college degree or higher (OR = 1.82; 95%CI = 1.30–2.56) than LOCRC patients. EOCRC cases were less likely to be former smokers compared to never smokers (OR = 0.50; 95%CI = 0.37–0.67), to have diabetes (OR = 0.31; 95%CI = 0.19–0.51), or to have a family history of CRC (OR = 0.60; 95%CI = 0.41–0.89) than those with LOCRC. Birth weight and pre-diagnostic adult BMI did not differ between the two groups.

### Stratified and sensitivity analyses

We explored the impact of potential biases on our findings (Table 3). Because screening recommendations are based on age, we examined analyses with the sample restricted to those without prior CRC screening; results were consistent with those for the whole sample. The effect estimates for alcohol consumption and most alcohol consumption in one’s teens/twenties slightly decreased in magnitude compared to the main analysis.

**Table 3:** Comparisons of EOCRC to LOCRC restricted to those without prior history of CRC screening and without Lynch syndrome or a family history of CRC (likely sporadic cases)

Risk factors/symptoms	Main analyses^a^	Restricted to those without prior screening^a^	Restricted to those without a family history of CRC or Lynch syndrome^a^
	*N* = 1,306	*N* = 747	*N* = 1,043
	OR (95%CI)		
Binge drinking, during decade with most consumption (vs. never)^b,c^	3.22 (2.37, 4.40)	2.31 (1.60, 3.34)	3.26 (2.30, 4.61)
Binge drinking, during year before CRC diagnosis (vs. never)^b,c^	3.15 (2.31, 4.30)	2.13 (1.47, 3.09)	2.86 (2.02, 4.06)
Most alcohol intake in teens/twenties (vs. other decades)	1.85 (1.36, 2.51)	1.69 (1.17, 2.45)	1.89 (1.34, 2.66)
Alcohol intake ever (vs. never)	2.47 (1.55, 3.91)	1.91 (1.12, 3.28)	2.79 (1.63, 4.77)
Smoking status, current (vs. never)	0.99 (0.62, 1.58)	0.92 (0.54, 1.57)	0.82 (0.47, 1.45)
Smoking status, former (vs. never)	0.50 (0.37, 0.67)	0.62 (0.43, 0.88)	0.49 (0.35,0.68)
Birth weight, > 10 lbs (vs. 6-8lbs)^c^	0.60 (0.23, 1.60)	0.51 (0.17, 1.58)	0.62 (0.21, 1.88)
Birth weight, 8–10 lbs (vs. 6-8lbs)^c^	1.00 (0.70, 1.42)	1.01 (0.67, 1.52)	1.10 (0.75, 1.62)
Birth weight, < 6 lbs (vs. 6-8lbs)^c^	0.84 (0.51, 1.38)	0.78 (0.42, 1.43)	0.81 (0.46, 1.43)
Inflammatory bowel disease (yes vs. no)^d^	1.13 (0.50, 2.58)	0.56 (0.06, 5.07)	0.84, (0.31, 2.30)
Irritable bowel syndrome (yes vs. no)^d^	0.92 (0.49, 1.75)	1.31 (0.50, 3.42)	0.88 (0.42, 1.81)
Diabetes (yes vs. no)^d^	0.31 (0.19, 0.51)	0.42 (0.23, 0.74)	0.31 (0.19, 0.53)
Lynch syndrome (yes vs. no)^d^	4.61 (2.72, 7.84)	4.64 (2.18, 9.91)	–
Family history of CRC (yes vs. no)	0.60 (0.41, 0.89)	0.54 (0.32, 0.92)	–
BMI pre-diagnosis (per 10 unit change)	1.13 (0.92, 1.39)	1.09 (0.86, 1.39)	1.21 (0.96, 1.52)
Any CRC symptoms pre-diagnosis (yes vs. no)	6.08 (3.77, 9.82)	7.22 (3.87, 13.47)	9.17 (4.89, 17.22)
Education, college degree or higher ( vs. ≤ HS degree)	1.82 (1.30, 2.56)	2.22 (1.47, 3.34)	1.93 (1.31, 2.83)
Race, non-white/multi-race (vs. white)	1.34 (0.83, 2.17)	1.33 (0.74, 2.38)	1.40 (0.82, 2.41)
Sex, Female (vs. male)	1.18 (0.90, 1.55)	1.24 (0.90, 1.71)	1.29 (0.95, 1.74)

OR = odds ratio; 95%CI = 95% confidence intervals; lbs = pounds; HS = high school

^a^Models adjusted for race, sex, education, BMI, smoking status, and family history of CRC. When a covariate was the independent variable in the model or used to restrict the sample, it was not treated as a confounder or adjusted for

^b^Binge drinking was defined as consuming ≥ 4 alcoholic drinks in a 2-hour period based on the OCCPI questionnaire

^c^Sample sizes for birth weight analyses: main analysis *N* = 1,107; restricted to prior CRC screening *N* = 643; and restricted to those without family history or Lynch Syndrome *N* = 894. Sample Sizes for binge drinking analyses: Main analysis, binge drinking 1-yr before CRC DX *N* = 1,297 & binge drinking during decade with highest alcohol consumption *N* = 1,269; restricted to prior CRC screening, binge drinking 1-yr before CRC DX *N* = 728 & binge drinking during decade with the most alcohol consumption *N* = 742; restricted to those without family history or Lynch Syndrome, binge drinking 1-yr before CRC DX *N* = 1,036 & binge drinking during decade with the most alcohol consumption *N* = 1,013

^d^Self-reported disease status, “Has a provider ever told you that you had the following conditions?”

In analyses restricted to likely sporadic cases, excluding those with a family history of CRC or Lynch syndrome, ever consuming alcohol and most alcohol consumption during one’s teens/twenties associations were stronger than in the total sample (OR = 2.79; 95% CI: 1.63–4.77 and OR = 1.89; 95%CI: 1.34–2.66, respectively). In likely sporadic EOCRC, the association with reporting CRC symptoms before diagnosis was greater compared to in the entire sample (OR = 9.17; 95%CI: 4.89–17.22).

We also compared risk factors across four age-at-diagnosis categories (i.e., < 40, 40–50, 50–65, ≥ 65 years) in Table 4. Seventy-one participants were diagnosed with CRC before age 40 (mean age 34.4) and 505 were diagnosed after 65 (mean age 72.4). Those < 40 years old were more likely to have been < 6lbs at birth (OR = 2.15; 95%CI: 0.95–4.87) compared to those ≥ 65. All alcohol related associations were greater in magnitude when the youngest EOCRC were compared to the oldest LOCRC cases.

**Table 4:** Baseline participant characteristics: four categories of age at diagnosis, Ohio Colorectal Cancer Prevention Initiative (*N* = 1,306)

Risk factors/symptoms	Age at diagnosis, years				*p*-value^a^	Crude OR (95%CI)
	< 40	40–50	50–65	≥ 65		
	*N* = 71	*N* = 217	*N* = 513	*N* = 505		
	No. (%)					
Age at diagnosis, mean ± SD	34.4 ± 4.5	45.6 ± 2.7	57.0 ± 4.4	72.4 ± 6.1	< .0001	─
Sex					0.205	
Female	33 (46.5)	122 (56.2)	256 (49.9)	242 (47.9)		0.94 (0.57–1.55)
Male	38 (53.5)	95 (43.8)	257 (50.1)	263 (52.1)		ref
Educational attainment					0.002	
≤ High school graduate	20 (28.2)	62 (28.6)	188 (36.7)	214 (42.4)		ref
Some college/ technical school/associate degree	23 (32.4)	78 (35.9)	171 (33.3)	171 (33.9)		1.44 (0.77–2.71)
≥ College degree	28 (39.4)	77 (35.5)	154 (30.0)	120 (23.8)		2.50 (1.35–4.62)
Insurance status^b^					0.001^f^	
Insured	71 (100)	212 (97.7)	502 (97.9)	505 (100)		─
Uninsured	0	5 (2.3)	11 (2.1)	0		─
Race					0.611	
White	65 (91.6)	197 (90.8)	477 (93.0)	472 (93.5)		ref
Non-whtie or Multi-race	6 (8.5)	20 (9.2)	36 (7.0)	33 (6.5)		1.32 (0.53–3.27)
BMI, (kg/m^2^), mean ± SD	29.2 ± 6.6	29.5 ± 6.5	29.8 ± 7.0	28.3 ± 5.3	0.001	1.27 (0.54–1.90)
Pre-diagnostic symptoms					< .0001^f^	
Yes	68 (95.8)	200 (92.2)	361 (70.4)	348 (68.9)		10.22 (3.17–33.0)
Family history of CRC					0.057	
Yes	6 (8.5)	29 (13.4)	98 (19.1)	90 (17.8)		0.43 (0.18–1.01)
Lynch syndrome					< .0001	
Yes	10 (14.1)	23 (10.6)	29 (5.7)	8 (1.6)		10.19 (3.88–26.80)
Diabetes^b^					< .0001^f^	
Yes	5 (7.0)	17 (7.8)	80 (15.6)	122 (24.2)		0.24 (0.09–0.60)
High cholesterol^b^					< .0001	
Yes	8 (11.3)	35 (16.1)	171 (33.3)	269 (53.3)		0.11 (0.05–0.24)
Irritable bowel syndrome^b^					0.665^f^	
Yes	4 (5.6)	9 (4.2)	22 (4.3)	29 (5.7)		0.98 (0.33–2.88)
Inflammatory bowel disease^b^					0.754^f^	
Yes	2 (2.8)	6 (2.8)	15 (2.9)	10 (2.0)		1.44 (0.31–6.69)
Birth weight^c^					0.182	
< 6 lbs	10 (14.5)	17 (8.0)	65 (12.4)	39 (7.7)		2.15 (0.95–4.87)
6–8 lbs	38 (55.1)	148 (69.5)	317 (60.6)	339 (66.5)		ref
8–10 lbs	20 (29.0)	43 (20.2)	124 (23.7)	115 (22.6)		1.70 (0.93–3.13)
> 10 lbs	1 (1.5)	5 (2.4)	17 (3.3)	17 (3.3)		0.53 (0.07–4.10)
Smoking status					< .0001	
Never	45 (63.4)	133 (61.3)	243 (47.4)	225 (44.6)		ref
Current	6 (8.5)	24 (11.1)	62 (12.1)	35 (6.9)		0.86 (0.34–2.16)
Former	20 (28.2)	60 (27.7)	208 (40.6)	245 (48.5)		0.41 (0.23–0.71)
Alcohol consumption					< .0001	
Ever consumed	63 (88.7)	200 (92.2)	462 (90.1)	394 (78.0)		2.22 (1.03–4.77)
Never consumed	8 (11.3)	17 (7.8)	51 (9.9)	111 (22.0)		ref
Decade in life with the most alcohol consumption^d^					< .0001	
Teens/twenties	54 (85.7)	131 (65.5)	301 (65.4)	179 (45.8)		7.11 (3.41–14.79)
Other decades	9 (14.3)	69 (34.5)	159 (34.6)	212 (54.2)		ref
Binge drinking, during decade with most consumption^e^					< .0001	
Ever engaged	47 (66.2)	128 (61.2)	270 (54.1)	150 (30.6)		4.44 (2.62–7.53)
Never engaged	24 (33.8)	81 (38.8)	229 (45.9)	340 (69.4)		ref
Binge drinking, during year before CRC diagnosis^e^					< .0001	
Ever engaged	32 (45.1)	76 (35.4)	131 (25.7)	55 (11.0)		6.65 (3.86–11.48)
Never engaged	39 (54.9)	139 (64.7)	379 (74.3)	446 (89.0)		ref

EOCRC = early-onset colorectal cancers, < 50 years; LOCRC = late-onset colorectal cancers, ≥ 50 years; OR = odds ratio; 95%CI = 95% confidence interval; SD = standard deviation; lbs = pounds

^a^*p*-values generated from chi-square tests or fisher exact tests^f^ (categorical variables) or Anova’s (continuous variables); calculations do not include missing values

^b^Self-reported disease status, “Has a provider ever told you that you had the following conditions?”

^c^Did not exclude those with missing birth weight data from the complete-case sample due to a large amount of missing in this variable, *N* = 1,107

^d^Lifetime alcohol abstainers were not included in this analysis (*N* = 182)

^e^Binge drinking was defined as consuming ≥ 4 alcoholic drinks in a 2-hour period based on the OCCPI questionnaire. Sample sizes for Binge drinking, during decade with most consumption *N* = 1,269; Binge drinking, during year before CRC DX *N* = 1,297

## Discussion

In this case-case comparison of EOCRC and LOCRC, ever consuming alcohol, consuming more alcohol in one’s teens/twenties, binge drinking, and Lynch syndrome were more associated with EOCRC than with LOCRC. The above-mentioned associations increased in magnitude when the sample was restricted to likely sporadic EOCRC cases, those without a family history of CRC or Lynch syndrome, indicating that alcohol consumption may be more relevant in sporadic EOCRC cases. Further, the associations with alcohol were stronger when comparing the youngest cases with the oldest. As expected, EOCRC cases were more likely to report experiencing pre-diagnostic CRC symptoms than those with LOCRC, because of screening age guidelines [26]. No association was observed between birth weight and EOCRC when compared to LOCRC. While not statistically significant, in a sensitivity analysis the youngest EOCRC cases, < 40 years old, were more likely to be born < 6lbs than the oldest LOCRC cases, those ≥ 65 (OR= 2.15; 95%CI: 0.95–4.87, unadjusted). Our study identified several factors that warrant further exploration as potential targets for primary or secondary prevention efforts.

Those with EOCRC were more likely to have ever consumed alcohol (OR= 2.50; 95%CI: 1.57–3.96) and to have a history of consuming the most alcohol in their teens and twenties (OR= 1.84; 95%CI: 1.35–2.49) compared to those with LOCRC. Binge drinking, whether during the decade of life with the most alcohol consumption or during the year before CRC diagnosis, was more associated with EOCRC than LOCRC (OR= 3.22; 95%CI: 2.37–4.40 and OR= 3.15; 95%CI: 2.31–4.30, respectively). We had concerns that younger individuals were not yet able to consume alcohol in “later life” as they had not reached that age. As a solution, we explored the decade of most alcohol consumption in teens/twenties (decades that all participants had experienced) compared to all other decades. Next, to focus on likely sporadic CRC cases, we restricted the analysis to those without Lynch syndrome or a family history of CRC. Results remained largely unchanged with one exception. After restricting to sporadic CRC, associations with ever using alcohol and most alcohol consumption in their teens/twenties increased in magnitude. Lastly, when comparing the youngest EOCRC to the oldest LOCRC cases, all alcohol effect estimates increased in magnitude, indicating more pronounced differences in alcohol consumption.

Our findings suggest a hypothesis-generating pathway linking alcohol consumption among sporadic EOCRC cases, which requires verification. Observations could be a result of other factors including cohort effects. Alcohol and its metabolite, acetaldehyde, are known carcinogens[27] and established risk factors for CRC [27]. Our findings are consistent with a meta-analysis showing that consuming alcohol was associated with a 71% increase in risk of developing EOCRC compared to never drinkers [17]. Other researchers explored alcohol consumption and risk of CRC finding that the association was stronger for EOCRC than LOCRC [28]. However a case-case comparison study conducted in Taiwan found that those with EOCRC were less likely to ever consume alcohol compared to LOCRC cases [29]. These authors were unable to conduct adjusted regression analyses and did not have alcohol exposure data as detailed as in our study. In the United States, per capita ethanol consumption increased starting around 1995 (2.1 gallons of ethanol) until the end of follow-up in 2019 (2.5 gallons of ethanol) [30]. It is unclear why there are differences in alcohol associations by age at diagnosis. It may be that there is a difference in the etiology for EOCRC or it may be differences in overall drinking patterns in younger individuals, such as consuming more alcohol, engaging in binge drinking more often, or starting to drink at a younger age. The 2025 Surgeon General’s Report on Alcohol and Cancer, has called for more research on how binge drinking and drinking at different developmental periods (i.e., adolescence) may impact cancer risk [31]. Our study’s findings highlight the need for continued research in this area as we see signals that both binge drinking and drinking patterns during young adulthood may play a role in EOCRC development.

There was nearly four-fold higher odds of EOCRC patients being diagnosed with Lynch syndrome than LOCRC cases, consistent with what is known [14, 32]. Similarly we found that the reporting of blood in stool, change in bowel habits, and bowel obstruction were more likely among EOCRC cases than LOCRC cases, again consistent with previous studies and no routine screening of younger patients [33–35]. Both Lynch syndrome and the reporting of CRC symptoms are factors that may aid in the identification of high risk younger individuals. There have been calls for the recognition of CRC symptoms in younger individuals among physicians as well as the general public to assist in earlier diagnosis [33].

We observed no differences between EOCRC and LOCRC by sex, race/ethnicity, IBS, or IBD. Diabetes, family history of CRC, or being a former smoker were less associated with EOCRC than LOCRC in this case-case comparison. It is important to note that these clinical factors may be associated with EOCRC risk, when compared to a disease-free group of same aged individuals rather than those with LOCRC. For example, increasing age and risk of diabetes status are positivity correlated, which may explain why LOCRC cases were more likely to be diabetic [36]. Different from our findings, male sex, IBD, family history of CRC, and hyperlipidemia were more prevalent among EOCRC cases when compared to same aged controls or LOCRC cases in other studies [17, 20, 37].

Birth weight is a proxy for accumulated fetal exposures, a potential critical window of susceptibility to exposure [19]. Because of the closer proximity in time between birth and diagnosis, the impact of early life exposures may be stronger in EOCRC cases [19]. It has been hypothesized that babies born heavier than average may be at increased risk of cancer because of higher exposure to in utero insulin-like growth factor I (IGF-I) [19]. In LOCRC cases, birth weight has been found to be minimally associated with overall risk of colon but not rectal cancer [38, 39]. To our knowledge, no other studies have examined birth weight and EOCRC. While we observed no statistically significant differences in birth weight when using a cut point of 50 years, there was a higher prevalence of low birth weight among the youngest EOCRC (< 40) compared to the oldest LOCRC cases (≥ 65 years). Birthweight averages have changed over time; between the 1960s and the 1990s birthweights increased, before decreasing again in 2005 [40]. Therefore, our findings may be, at least in part, a reflection of secular trends in exposure [40]. It is unclear if or how birthweight, potentially low birth weight, may be contributing to EOCRC etiology.

### Strengths and limitations

This study has several strengths. First, by utilizing data from across the state of Ohio, including participants from rural and urban communities, our study likely approaches a more population-based sample than prior studies. Previous studies of specific risk factors and EOCRC have been conducted in special populations (i.e., women only, veterans) [41, 42] or at a single-center [20]. Nonetheless, this sample did not include all sites throughout the state and there could be some selection pressures. Another strength is the number of EOCRC cases included (*N* = 323 total); several prior studies had smaller samples of EOCRC cases than ours [15, 41, 41, 43]. Additionally, the case-case study design helps to mitigate concerns of recall bias because the entire sample had been recently diagnosed with CRC, compared to a case-control study design.

This study has important limitations that should be considered when interpreting the findings. The case-case design is primarily hypothesis generating, allowing for identification of differences in risk factors between EOCRC and LOCRC, but not associations with risk of developing EOCRC. Without an age-matched control group, our effect estimates observed are strongly influenced by the prevalence of risk factors in the two groups because of their age. Selection biases may have been introduced due to inclusion criteria of the OCCPI (i.e., tumor tissue availability and completion of the questionnaire). The exclusion of participants with missing covariate data did not meaningfully skew the study population: the original sample was comprised of 21% EOCRC and 80% LOCRC cases; after excluding those with missing data the proportion remained similar, 22% EOCRC and 78% LOCRC cases. Similarly, screening at the population level in asymptomatic adults was historically recommended to start at age 50. In 2021 the United States Preventive Services Task Force recommendation changed to include those 45–50 years old [22]. The OCCPI was conducted before 2021; the majority of EOCRC cases would have not been detected by routine screening, unless they were considered high-risk (i.e. CRC family history, IBD, or symptoms severe enough to result in testing). We explored potential outcome detection bias by restricting the sample to those without prior CRC screening. The findings were similar. Our sample was predominantly white and medically insured, which may impact the generalizability of the findings. However, the EOCRC cases in OCCPI had biologically similar tumors to other populations of EOCRC, with the majority presenting in the left colon or rectum (Supplemental Materials, Table 3) [20, 32, 44].

We found that alcohol consumption was more associated with EOCRC than LOCRC including ever consuming alcohol, consuming the most alcohol in one’s teens/twenties, and binge drinking. All of these associations were stronger among those who did not report a family history of CRC, suggesting that alcohol may play a role particularly for non-familial cases. These exploratory findings generate important hypotheses regarding the role of alcohol consumption in EOCRC, which may ultimately help identify high-risk younger individuals who may benefit from targeted CRC screening. Finally, these findings reinforce the need for public health messaging regarding CRC symptoms to include younger adults.

## Supplementary Information

Below is the link to the electronic supplementary material.

Supplementary file1 (DOCX 26 KB)

## Data Availability

The OCCPI makes data accessible for re-use by other investigators. An investigator is required to submit an access request and proposal to the OCCPI investigators for approval.
