# Supplementary material for: Early and late-onset colorectal cancers: a case-case comparison of risk factors
Source: Cancer Causes Control. 2026 Jul 11;37(8):126. doi: 10.1007/s10552-026-02208-2 (PMC13356065; doi:10.1007/s10552-026-02208-2)
Supplement: Supplementary file 1 — Supplementary file1 (DOCX 26 KB) [file 10552_2026_2208_MOESM1_ESM.docx]

**Supplemental Materials**

| ***Supplemental Table 1.*** Risk factor assessment in the OCCPI epidemiologic questionnaire. | | |
| --- | --- | --- |
| **Risk Factor** | **Assessment** | **Response Options** |
| Clinical conditions (i.e., diabetes, irritable bowel syndrome (IBS), inflammatory bowel disease (IBD), hypertension) | “Has a doctor or other healthcare provider ever told you that you had any of the following conditions or illnesses?” | Yes, No, I don’t know |
| Birth weight |  | Less than 6 pounds, 6-8 pounds, 8-10 pounds, more than 10 pounds, or I don’t know” |
| Height and weight | Prior to your recent colorectal cancer diagnosis, what was your average weight as an adult?” and “How tall are you?” | Open-ended response |
| Pre-diagnositc CRC symtpoms | What symptoms did you experience that led to your colorectal cancer diagnosis? Select all that apply” | Blood in stool, change in bowel habits, bowel obstruction, bowel perforation, anemia, weight loss, no symptoms, and I don’t know. *Pain was inconsistently assessed and therefore was not examined. |
| Alcohol Intake | **Ever Consuming Alcohol**, “Have you ever consumed beverages containing alcohol during your lifetime?”; **Binge Drinking Year Before Diagnosis**, “During the past year/year before your colorectal cancer diagnosis, how often did you have 4 or more drinks containing any kind of alcohol within a two-hour period?; **Decade in Life with Most Consumption**, “During your lifetime, what time period did you drink the most alcohol?”; **Binge Drinking During Decade with Most Consumption**, “At the time in your life when you were drinking the most alcohol, how often did you have 4 or more drinks containing any kind of alcohol within a two-hour period?” | Yes, No, I don’t know  How Often: Every day, 4-6 times per week,1-3 times per week, A few times per month, Once a month, Less than once a month, Never, I don't know |
| Smoking Status | Have you smoked at least 100 cigarettes during your lifetime?” and “Do you currently smoke cigarettes?”. | Yes, No, Unknown |

| ***Supplemental Table 2.*** Associations between potential risk factors and EOCRC vs. LOCRC, using different confounder adjustment methods (model-specific or same set confounder adjustment). | | | |
| --- | --- | --- | --- |
| **Risk Factor** | **Model-Specific Confounders** | **Model-Specific Adjustment** | **Same Set Confounder Adjustment**^a^ |
|  |  |  | *(presented in the main text)* |
|  |  | Odds Ratio (95%CI) | |
| **Sex,** Female (vs. male) | BMI, smoking status, alcohol ever | 1.21 (0.93, 1.59) | 1.18 (0.90, 1.55) |
| **Education,** College Degree or Higher (vs. ≤ HS degree) | sex, BMI, smoking status, alcohol ever, diabetes status | 1.55 (1.09, 2.19) | 1.82 (1.30, 2.56) |
| **Race,** non-White/Multi-race (vs. white) | smoking status, alcohol ever, diabetes status, any symptoms | 1.47 (0.89, 2.43) | 1.34 (0.83, 2.17) |
| **BMI Pre-Diagnosis** (per 10 unit change) | sex, smoking status, alcohol ever, diabetes status | 1.24 (1.00, 1.54) | 1.13 (0.92, 1.39) |
| **Any CRC Symptoms Pre-Diagnosis** (yes vs. no) | education, sex, diabetes status, BMI, IBD | 6.03 (3.74, 9.74) | 6.08 (3.77, 9.82) |
| **Family History of CRC** (yes vs. no) | education, sex, Lynch Syndrome | 0.50 (0.33, 0.75) | 0.60 (0.41, 0.89) |
| **Lynch Syndrome** (yes vs. no) | race and family history of CRC | 4.23 (2.53, 7.05) | 4.61 (2.72, 7.84) |
| **Diabetes** (yes vs. no) ^b^ | education, sex, race, BMI | 0.32 (0.20, 0.52) | 0.31 (0.19, 0.51) |
| **Irritable Bowel Syndrome** (yes vs. no) ^b^ | sex and race | 0.87 (0.46, 1.62) | 0.92 (0.49, 1.75) |
| **Inflammatory Bowel Disease** (yes vs. no) ^b^ | sex and prior screening | 1.94 (0.84, 4.50) | 1.13 (0.50, 2.58) |
| **Birth Weight**, <6 lbs (vs. 6-8lbs)b | sex and race | 0.85 (0.52, 1.38) | 0.84 (0.51, 1.38) |
| **Smoking Status,** Current (vs. never) | education, sex, alcohol ever | 0.82 (0.52, 1.30) | 0.99 (0.62, 1.58) |
| **Alcohol Intake Ever** (vs. never) | education, sex, smoking status | 2.50 (1.57, 3.96) | 2.47 (1.55, 3.91) |
| **Most Alcohol Intake in Teens/Twenties** (vs. other decades) | education, sex, smoking status | 1.84 (1.35, 2.49) | 1.85 (1.36, 2.51) |
| **Binge Drinking,** During Year Before CRC DX (vs. never)^a^* | education, sex, smoking status | 3.08 (2.26, 4.19) | 3.15 (2.31, 4.30) |
| **Binge Drinking,** During Decade with Most Consumption (vs. never)^a^* | education, sex, smoking status | 3.22 (2.37,4.39) | 3.22 (2.37, 4.40) |
| **Notes:** EOCRC=early-onset colorectal cancers, <50 years ; LOCRC=late-onset colorectal cancers, ≥ 50 years | | | |
| ^a^Models adjusted for race, sex, education, BMI, smoking status, and family history of CRC. When a covariate was the independent variable in the model or used to restrict the sample then it was not treated as a confounder or adjusted for. | | | |
| ^b^Self-reported disease status, “Has a provider ever told you that you had the following conditions?”. | | | |

| ***Supplemental Table 3.*** Tumor and treatment characteristics of EOCRC and LOCRC cases. | | | |
| --- | --- | --- | --- |
| Characteristics | **Early-onset CRC** (n=323) | **Late-onset CRC** (n=1,256) | p-value^a^ |
|  |  |  |  |
|  |  |  |  |
|  | No. (%) | |  |
| **Tumor Site** |  |  | <.0001 |
| Right Colon | 68 (21.1) | 446 (35.5) |  |
| Traverse Colon | 16 (5.0) | 94 (7.5) |  |
| Left Colon | 100 (40.0) | 283 (22.5) |  |
| Rectum/Rectosigmoid | 124 (38.4) | 357 (28.4) |  |
| Not Otherwise Specified | 15 (4.6) | 76 (6.1) |  |
| **Cancer Treatment^b^** |  |  |  |
| Surgery |  |  | 0.24 |
| No | 36 (11.2) | 113 (9.0) |  |
| Yes | 287 (88.9) | 1,143 (91.0) |  |
| Radiation Therapy |  |  | 0.01 |
| No | 219 (67.8) | 951 (75.7) |  |
| Yes | 84 (26.0) | 248 (19.8) |  |
| *Missing or unknown* | 20 (6.2) | 57 (4.5) |  |
| Chemotherapy |  |  | <.0001 |
| No | 59 (18.3) | 472 (37.6) |  |
| Yes | 243 (75.2) | 716 (57.0) |  |
| *Missing or unknown* | 21 (6.5) | 68 (5.4) |  |
| Multiple Therapies |  |  | <.0001 |
| No | 70 (21.7) | 495 (39.4) |  |
| Yes | 232 (71.8) | 694 (55.3) |  |
| *Missing or unknown* | 21 (6.5) | 67 (5.3) |  |
| **Notes:** EOCRC=early-onset colorectal cancers, <50 years ; LOCRC=late-onset colorectal cancers, ≥50 years | | | |
| ^a^P-values were generated from a chi-square test. P-value calculations do not include missing values. | | | |
| ^b^For treatments (surgery, chemotherapy, or radiation) “yes”, indicates either that treatment was received or planned to receive at the time of the questionnaire. | | | |
